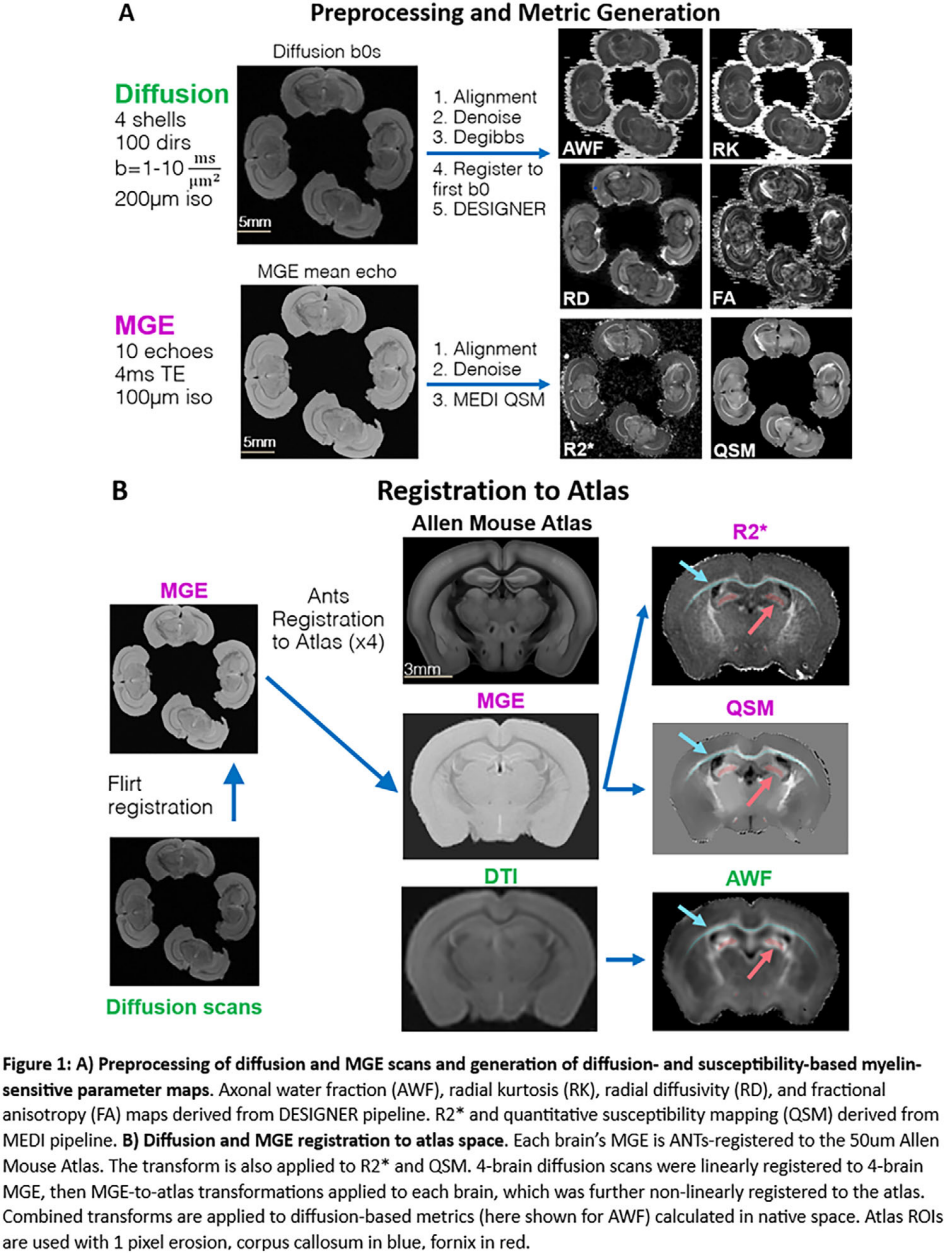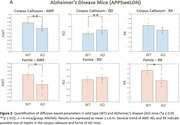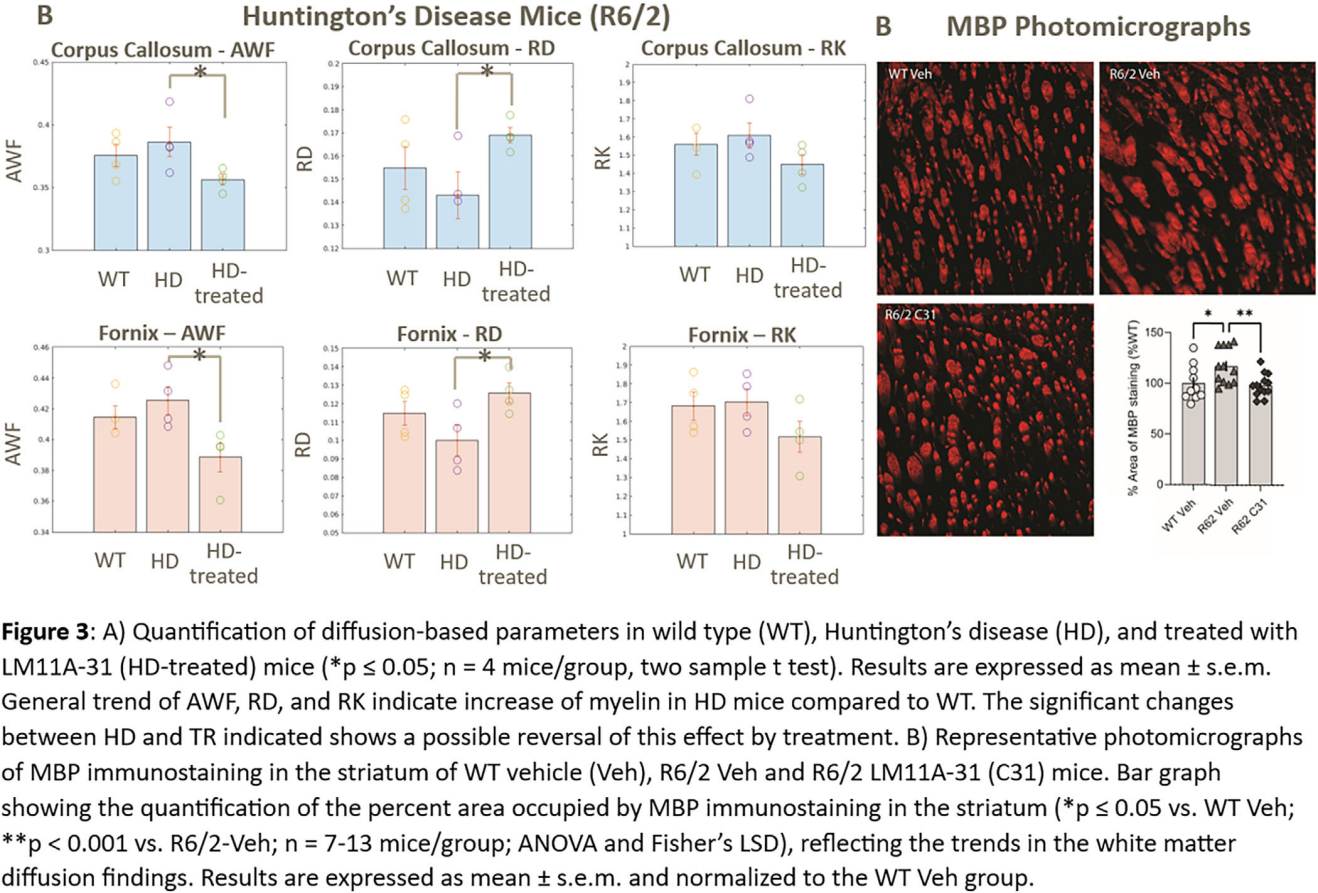# White Matter Changes in Alzheimer’s and Treated Huntington’s Disease Mouse Models Detected by Diffusion MRI

**DOI:** 10.1002/alz.093659

**Published:** 2025-01-09

**Authors:** Andy Liu, Danielle Simmons, Connor Alvarez, Yi Wang, Pascal Spincemaille, Frank M. Longo, Michael Zeineh, Marios Georgiadis

**Affiliations:** ^1^ Stanford University, Stanford, CA USA; ^2^ Weill Cornell Medicine, New York, NY USA; ^3^ Weill Cornell Medicine, Ithaca, NY USA

## Abstract

**Background:**

Myelin integrity is central to healthy brains and is increasingly shown to be compromised in neurodegenerative diseases. Diffusion‐ and susceptibility‐based MRI metrics can detect myelin changes. We show advanced diffusion and susceptibility metrics can detect degenerative myelin changes in ex vivo AD and HD mouse models.

**Method:**

AD mice included 2 groups: 8‐month‐old male hAPP (Lond/Swe mutations, n = 4) and wild‐type (WT, n = 4). HD mice included 3 groups: 12‐week‐old vehicle‐treated controls (WT, n = 4), R6/2 HD treated for 7 weeks with vehicle (HD, n = 4) or LM11A‐31 (HD‐treated, n = 4). After perfusion‐fixation and brain extraction, 4‐shell diffusion (b = 1,2,5,10ms/µm2, 100 directions) and multi‐echo gradient echo (MGE) MRI (10 echoes, GRE = 4‐40ms) were performed on a Bruker 7T scanner. We used DESIGNER and MEDI to generate diffusion‐ and susceptibility‐based myelin‐sensitive metrics, respectively (diffusion: mean/radial/axial diffusivities ‐MD/RD/AD, kurtoses ‐MK/RK/AK, axonal water fraction ‐AWF; susceptibility: R2*, quantitative susceptibility mapping‐QSM) (Fig. 1A). Datasets were registered to the Allen Atlas through linear/non‐linear transformations using FSL flirt/ANTs (Fig. 1B). Median values were derived for two Allen Atlas white matter regions‐of‐interest ‐ROIs (corpus callosum and fornix). For AD, scan timing effects were included in a 2‐way ANOVA analysis. For HD, two‐sample t‐tests were performed between the 3 groups. For histological validation, a second set of R6/2 HD brains (WT, HD, HD‐treated n = 10‐13 mice/group) were myelin basic protein (MBP)‐stained, and the percent area of striatum immunostaining determined by ImageJ thresholding.

**Results:**

AD mice showed increased RD and reduced AWF and RK compared to controls in both ROIs, suggesting a loss of myelinated axons. Susceptibility‐based metrics show no clear pattern. HD mice showed an increasing trend in AWF and RK and decreases in RD in both ROIs vs WT, suggesting increased myelination. Interestingly, LM11A‐31‐treated HD mice showed a significant reversal in RD and AWF. Histologically, myelin immunostaining was increased in the striatum of HD mice, reversed by LM11A‐31.

**Conclusion:**

Diffusion MRI myelin‐sensitive metrics detected possibly compromised myelin in AD mouse white matter (corpus callosum and fornix). In HD mice, both diffusion MRI and histology showed a trend towards increased myelin, which was reversed by LM11A‐31 treatment.